# IG1, a Mansonone F Analog, Exhibits Antibacterial Activity against *Staphylococcus aureus* by Potentially Impairing Cell Wall Synthesis and DNA Replication

**DOI:** 10.3390/life12111902

**Published:** 2022-11-16

**Authors:** Xin Chen, Yueqiao Lin, Qianqian Gao, Shiliang Huang, Zihua Zhang, Nan Li, Xin Zong, Xuemin Guo

**Affiliations:** 1 School of Medicine, Foshan University, Foshan 528000, China; 2 Zhongshan School of Medicine, Sun Yat-sen University, Guangzhou 510630, China; 3 School of Pharmaceutical Sciences, Sun Yat-sen University, Guangzhou 510630, China; 4 College of Animal Sciences, Zhejiang University, Hangzhou 316021, China; 5 Meizhou People’s Hospital, Meizhou 514031, China; 6 Guangdong Provincial Key Laboratory of Precision Medicine and Clinical Translation Research of Hakka Population, Meizhou 514031, China

**Keywords:** mansonone, *Staphylococcus aureus*, antimicrobial activity, DNA replication

## Abstract

Infection caused by *Staphylococcus aureus*, especially methicillin-resistant *S. aureus* (MRSA), is very common in communities and hospitals, which poses a great challenge to human health. Therefore, increasing attention has been paid to finding effective antimicrobial agents. Mansonone F is a natural compound which has an oxaphenalene skeleton and anti-*S. aureus* activity, but its sources are limited and its synthesis is difficult. Thus, IG1, a C9-substituent mansonone F analog, was assessed for its activity against *Staphylococcus aureus* and its mechanism of action was investigated. Antimicrobial susceptibility assays showed that IG1 has strong antibacterial activity against *S. aureus*, including MRSA, with minimum inhibitory concentrations (MICs) ranging from 0.5 to 2 μg/mL, which were very close to those of vancomycin, and these changed little, even with an increase in the amount of the inoculum. To further explore the antibacterial properties of IG1, time–kill experiments were conducted. Compared with vancomycin and moxifloxacin, treatment with different concentrations of IG1 reduced the viability of organisms in a very similar manner and the reduction was not significant, which indicated that IG1 is a potentially strong anti-*S. aureus* agent. Finally, the antibacterial mechanism was analyzed, with flow cytometric analysis revealing that IG1 treatment resulted in a time-dependent decrease in the DNA content of *S. aureus*. Transmission electron microscopy (TEM) analysis showed that very few dividing cells could be found and the cell wall was damaged in the field of IG1-treated cells. These results indicate that IG1 is a potential new antibacterial agent against *S. aureus*, including MRSA.

## 1. Introduction

*S. aureus*, especially MRSA, is an important public health problem around the world, which can cause a variety of serious infectious diseases such as bacteremia, sepsis, osteomyelitis, and pulmonary infection, and which results in thousands of deaths annually [1]. Vancomycin has been the treatment of choice for MRSA for decades, but its prolonged use has reduced drug susceptibility and treatment failure rates have increased year by year. It is thus very urgent to research and explore effective and safe drugs against *S. aureus*, even MRSA and vancomycin-resistant MRSA.

Mansonone compounds represent a series of natural products with an *o*-quinone structure, mainly isolated from the heartwood of *Mansonia altissima* and *Ulmus glabra*. This type of compound is classified as phytoalexins, which are produced and accumulated in plants in response to bacterial and fungal infections, and which have been used to treat Dutch elm disease (DED). Mansonone F is a mansonone compound, consisting of an oxaphenalene skeleton and an ortho-naphthoquinone moiety which has been noted as a relatively novel structure that rarely exists in natural products [2,3,4]. Mansonone F has shown comprehensive pharmacological activities, such as antibacterial and anti-tumor activity [5]. However, its low content in nature, as well as the difficulty of synthesizing this compound, have limited its applications. Therefore, many studies have focused on developing an easier route to obtain mansonone F analogs and to optimize the structure to obtain analogs with more potent bioactivity. As expected, some of the mansonone F analogs, such as C6 analogs 8a and 14b, also possess significant activity against Gram-positive bacteria, particularly MRSA, with MICs against MRSA even smaller than those of vancomycin [6,7,8]. Given their unique structure and potential mechanisms of action, which are distinct from other known antibiotics used for anti-MRSA therapies, mansonone F and its analogs have been expected to be developed into novel antimicrobial agents against Gram-positive bacteria and to provide a solution to current resistance [8,9]. However, more assays are required in order to demonstrate the antimicrobial activity of mansonone F or its analogs against the clinical isolates. Two possible anti-MRSA mechanisms have been proposed, one involving the generation of cytotoxic superoxide radicals, and the other relating to the modification and inactivation of growth-related enzymes due to the attachment of certain nucleophiles of the enzymes to the enedione carbonyl of mansonone F [10], but there is as yet no direct evidence for this.

**Figure 1:**
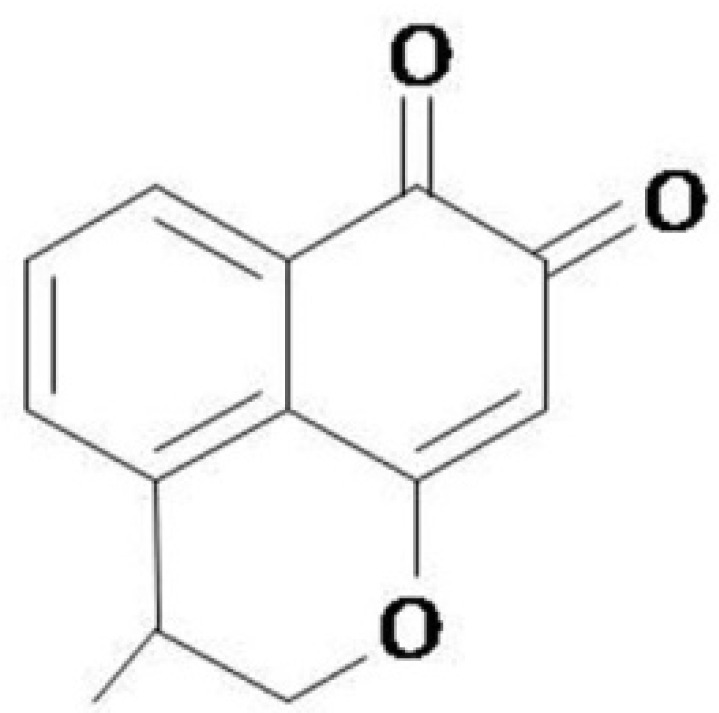
Structure of IG1.

Here, we report that IG1 (Figure 1), a C9 substituent mansonone F analog [11], showed significant activity against all the tested clinical isolates of *S. aureus*, including MRSA. Furthermore, its antimicrobial activity was further characterized via the time–kill assay. Meanwhile, its antibacterial mechanism was investigated, and propidium iodide (PI) staining combined with flow cytometric analysis was used to visualize the changes in the intracellular DNA content. Transmission electron micrography was used to indicate the state of cell division and the morphological changes in *S. aureus* after exposure to IG1. The results are expected to help uncover the antimicrobial mechanisms of IG1 and provide a potential new approach for the treatment of MRSA infection.

## 2. Materials and Methods

### 2.1. Regents

The mansonone F analog IG1 was designed and synthesized by professor Shiliang Huang [11]; vancomycin and moxifloxacin were obtained from the China Institute for Food and Drug Control, Beijing, China; and Luria–Bertani (LB) broth, LB agar and Mueller–Hinton (MH) broth were obtained from Oxoid, Basingstoke, UK. Propidium iodine (PI) was obtained from Thermo Scientific, Waltham, MA, USA, and RNase A was obtained from Takara, Maebashi, Japan.

### 2.2. Bacterial Identification and Culture

A total of 25 *S. aureus* isolates were collected at Meizhou People’s Hospital from March to October, 2020. The strain species were determined using a Vitek II system (bioMérieux, Durham) and further confirmed via PCR amplification of a *S. aureus*-specific chromosomal fragment as described previously [12]. These isolates showed different DNA patterns and thus belonged to different clone types based on random amplified polymorphic DNA polymerase chain reaction (RAPD-PCR) analysis with three different primers: 5′-GGTTGGGTGAGAATTGCACG-3′, 5′-GTGGATGCGA-3′ and 5′-AAGTAAGTGACTGGGGTGAGCG-3′ [13].

MH broth was used for antimicrobial susceptibility testing, LB broth and LB agar were used for time–kill assays, and bacterial cultures of *S. aureus* isolates were conducted under conditions of 37 °C.

### 2.3. Antimicrobial Susceptibility Testing

MICs of IG1 and vancomycin against all these isolates and the reference strain *S. aureus* ATCC 25923 were measured using the reference broth microdilution method at a standard inoculum of ~1 × 10^5^ CFU/mL and a high inoculum of ~1 × 10^9^ CFU/mL [14]. Briefly, ten successive dilutions of IG1 and vancomycin solution, which covered the antibacterial concentrations in the MH broth, were prepared in 1 to 10 columns of 96-well plates, then fresh *S. aureus* cultures in logarithmic phase were added to the target concentration. Columns 11 and 12 of the plates served as controls, the positive control was composed of the same concentration of bacteria, and the negative control was composed of the same volume of culture medium. Samples were then incubated at 37 °C for 18 to 24 h. MIC levels were defined as the lowest concentration of each antibiotic that completely inhibited the growth of the inoculum. The results were interpreted according to the Clinical and Laboratory Standards Institute (CLSI) guidelines [15].

### 2.4. Transmission Electron Microscopy Assay

For TEM assessments, MRSA strain 60035 in the early logarithmic phase was treated with 2 μg/mL IG1 for 4 h with shaking at 37 °C, then the cells were collected and fixed in 2% glutaraldehyde in 0.1 M sodium phosphate buffer (pH 7.4) for 4 h at 4 °C, followed by fixation with 1% osmium tetroxide for 2 h at 4 °C. Cells were dehydrated with graded concentrations of ethanol and embedded in Epon resin. Ultrathin sections were obtained using a diamond knife and embedded in a copper grid, then stained with uranyl acetate and lead citrate. Finally, the prepared samples were examined using TEM (JEM-1011, JEOL, Peabody, MA, USA). Digital electron micrographs were acquired with a 1024 × 1024-pixel CCD camera system (AMT Corp, Denver, MA, USA) [16,17].

### 2.5. Time–Kill Assays

The activities of IG1, vancomycin, and moxifloxacin against MRSA strain 60035 were evaluated by measuring the reduction in the numbers of CFU per milliliter over 2 h. Bacterial suspensions were prepared as described above. Fresh bacterium culture in logarithmic phase was diluted to a standard inoculum of ~1 × 10^5^ CFU/mL and a high inoculum of ~1 × 10^9^ CFU/mL, and the compounds were added to yield final concentrations of one, two, four, and eight times the respective MICs. The suspensions were mixed for 20 s with a vortex mixer, and a sample (0.5 mL) was removed at 30 s and plated onto LB agar plates after 10-fold serial dilutions. Additional samples were taken at 30, 60, and 120 min after incubation at 37 °C with shaking. When rapid killing occurred, samples were also taken at 3, 6, 9, 12, and 15 min. Samples were diluted, plated onto LB agar plates, and incubated overnight at 37 °C. The mean number of survivors was determined.

### 2.6. Measurement of DNA Content

Measurements of intracellular DNA content were performed using PI staining. MRSA strain 60035 in the early logarithmic phase was treated with 2 μg/mL IG1 and 2 μg/mL vancomycin for different times, respectively. Then, the cells were collected and fixed in absolute alcohol for 2 h and the cells were washed twice and resuspended in PBS. Then, 100 μg/mL RNase A was added and allowed to incubate for 1 h at 37 °C. PI was added at a final concentration of 0.05 mg/mL, following 0.5 h incubation, protected from light. DNA content was analyzed using a Coulter EPICS-XL flow cytometer (Beckman Coulter, Fullerton, CA, USA). Data analysis was carried out using EXP032 software.

### 2.7. Statistical Analysis

Any experiment that related to calculation had been executed separately at least three times and the results are given as the mean ± standard deviation. The statistical significance of the intracellular DNA level, comparing control and IG1-treated cells, was determined via Student’s *t*-test, using IBM SPSS Statistics for Windows v. 19.0.

## 3. Results

### 3.1. Antibacterial Activity of IG1 on S. aureus

**Figure 2:**
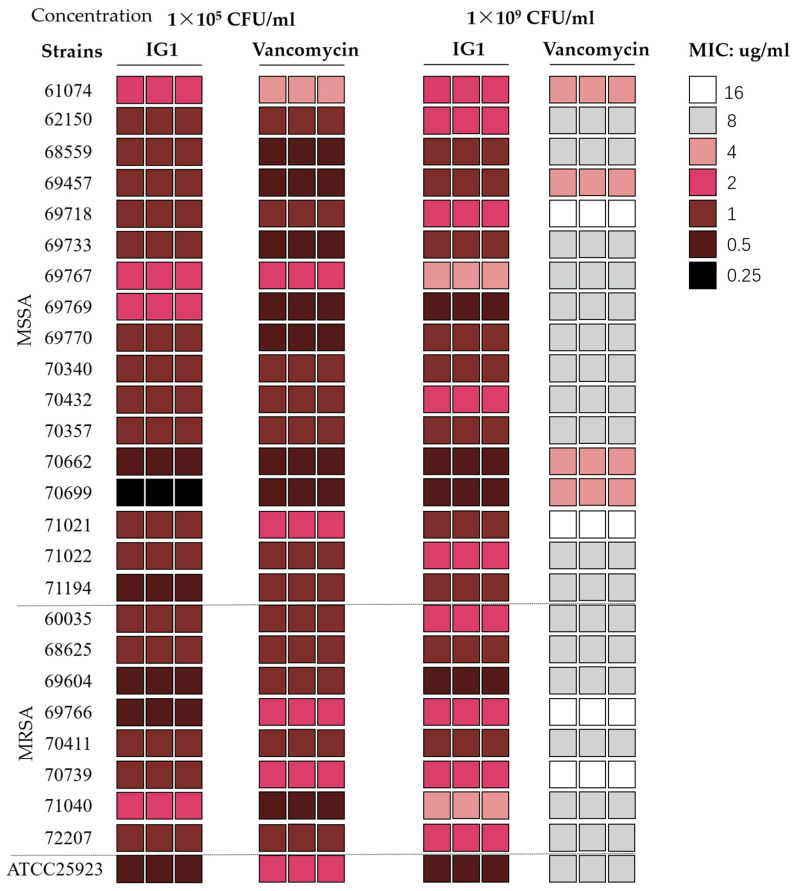
Schematic diagram of the broth microdilution method to evaluate the MICs of IG1 and vancomycin against *S. aureus* strains (tested in triplicate). The starting inoculation concentration was 1 × 10^5^ CFU/mL (left) and 1 × 10^9^ CFU/mL (right). Different colored squares represent different MICs. A total of 26 *S. aureus* strains, including 17 methicillin-susceptible *S. aureus* (MSSA) strains and 8 methicillin-resistant *S. aureus* (MRSA) strains and the reference strain ATCC25923, were tested.

The antibacterial activity of IG1 compared to that of vancomycin on 25 clinical *Staphylococcus aureus* isolates was evaluated through the determination of MIC values (Figure 2). Among the tested isolates, 17 (68%) were methicillin-susceptible *S. aureus* (MSSA) and 8 (32%) were MRSA. MIC determination results showed that all the tested isolates were susceptible to vancomycin, with MICs ranging from 0.5 to 2 μg/mL according to the Clinical and Laboratory Standards Institute (CLSI) guidelines [15]. The MIC values of IG1 for these tested isolates, whether methicillin-sensitive or methicillin-resistant, ranged from 0.25 to 2 μg/mL, very close to those of vancomycin and some MSSA isolates have MIC values even smaller than that of vancomycin (Table 1). Once the inoculum concentration of these isolates rose to 1 × 10^9^ CFU/mL, MIC values against IG1 did not or were only weakly altered, but changed significantly from 0.5~2 μg/mL to 4~16 μg/mL against vancomycin (Table 1).

### 3.2. Time–Kill Kinetics of IG1

**Figure 3:**
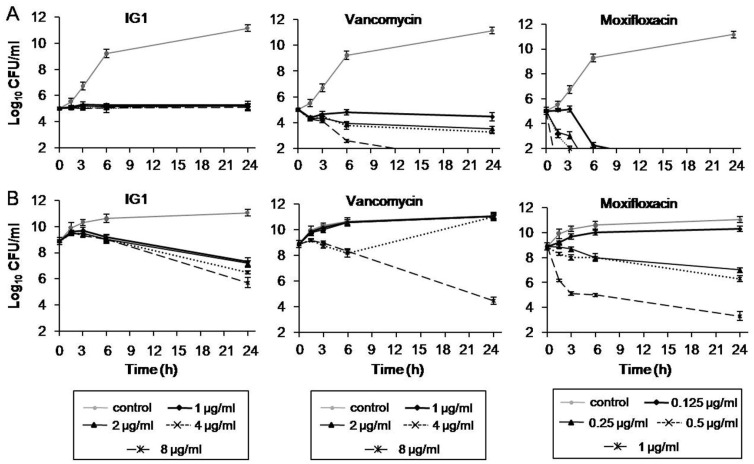
Time–kill assays examining the use of IG1 against *S. aureus* 60035 with vancomycin and moxifloxacin used as controls. The starting inoculation was 1 × 10^5^ CFU/mL (**A**) and 1 × 10^9^ CFU/mL (**B**). The agent concentrations were marked. The cultures were collected at 0, 1.5, 3, 6, and 24 h and subcultured to agar media without antibiotics; the number of colonies was counted and calculated after incubation. Data are means ± S.D. of three experiments.

The activity of IG1 against *S. aureus* was further evaluated by measuring the reduction in viable counts over 24 h via the time–kill methodology according to NCCLS guidelines [18]. The MRSA strain 60035 was randomly chosen for this assay. The time–kill kinetics of IG1, vancomycin and moxifloxacin were tested at the MIC values of 1, 1, and 0.125 μg/mL, respectively. At a standard inoculum of ~1 × 10^5^ CFU/mL, IG1 treatment at a concentration ranging from 1 to 8 μg/mL reduced the organisms’ viability in a very similar manner and the reduction was less than 1 log^10^ over 24 h (Figure 3A, left panel). In contrast, treatment with vancomycin or moxifloxacin at 8 μg/mL and 0.125 μg/mL, respectively, achieved 99.9% kill (greater than 3 log^10^ reductions) within 24 h (Figure 3A, middle and right panel). When the inoculum concentration rose from 1 × 10^5^ to 1 × 10^9^ CFU/mL, the time–kill behaviors of these antimicrobial agents changed to variable degrees. IG1 treatment at 1 and 8 μg/mL resulted in 1.2 log^10^ and 2.7 log^10^ reductions at 24 h, respectively, much more significant than the same treatment at low inoculation (Figure 3B, left panel). As expected, the treatment with vancomycin or moxifloxacin both clearly attenuated their inhibitory role on the viability at lower concentrations (MIC, 2 × MIC, and 4 × MIC) compared with standard inoculum, but still showed strong bactericidal activity at the concentration up to 8 and 1 μg/mL (8 × MIC), respectively (Figure 3B, middle and right panel).

### 3.3. Effect of IG1 on DNA Content and Cell Division of S. aureus

**Figure 4:**
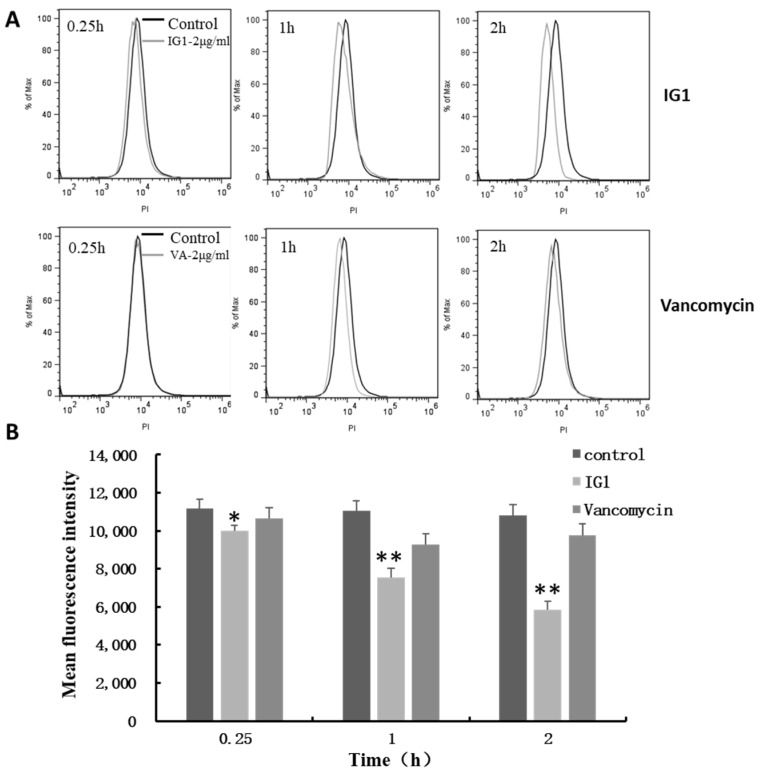
Effect of IG1 on the synthesis of the DNA content in *S. aureus*, cells were treated with IG and vancomycin for 0.25, 1, and 2 h, respectively. (**A**) Fluorescence intensity distribution of the samples, the black and grey curves represent the control cells and drug-treated cells, respectively. (**B**) Quantitative levels of intracellular DNA. The DNA value was expressed as mean fluorescence intensity. Data are means ± S.D. of three experiments. * represent *p* < 0.05 compared with control and ** represent *p* < 0.01 compared with control.

**Figure 5:**
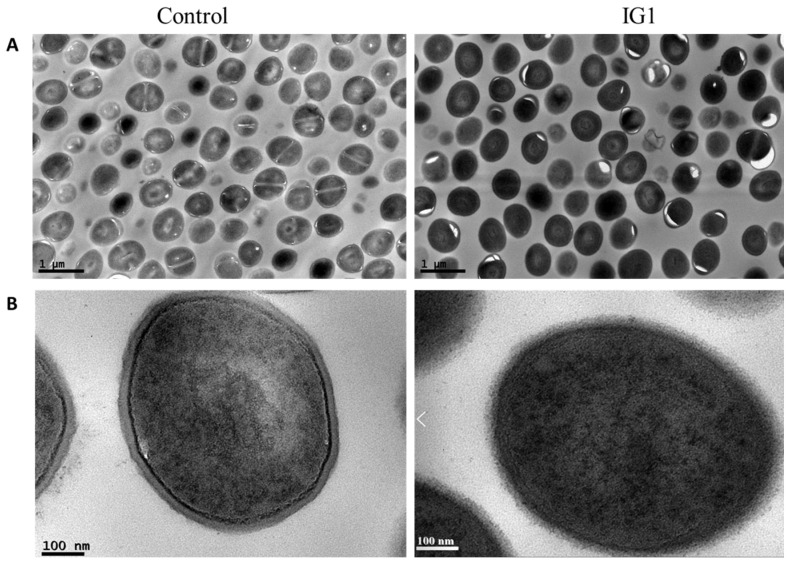
Transmission electron microscopy of MRSA strain 60035 without antimicrobial agent treatment (**left**) or with 2 μg/mL IG1 treatment for 4 h (**right**). (**A**) Multiple-cell image; bars represent 1 μm. (**B**) Individual cell microstructure; bars represent 100 nm.

To investigate whether the effect of IG1 was related to DNA replication, the intracellular DNA content was examined using fluorescent probe propidium iodide. The results showed that treatment of 2 μg/mL IG1 in the MRSA stain 60035 resulted in a time-dependent decrease in intracellular DNA content (Figure 4). In contrast, treatment with vancomycin at 2 μg/mL only showed a slight decrease at 1 h treatment and no further decrease was observed at the point of 2 h (Figure 4). Then, cell division status was examined using a transmission electron macrograph. MRSA stain 60035 in the early logarithmic phase was treated with IG1 at 2 μg/mL for 4 h, and untreated MRSA 60035 was used as control. The image of a randomly chosen field from each of the two samples was presented to show the state of cell division. Very few dividing cells could be found in the field of IG1-treated MRSA 60035 (Figure 5A, right). In contrast, nearly half of the cells were dividing in untreated 60035 (Figure 5A, left).

### 3.4. Effect of IG1 on Cell Morphology of S. aureus

The structure of *S. aureus* after exposure to IG1 was also examined using a transmission electron micrograph. MRSA strain 60035 in the logarithmic phase was treated with IG1 at 2 μg/mL for 4 h, and the cell suspension without another antimicrobial agent treatment was used as a control. An image of a randomly chosen cell from each of the two samples is presented to illustrate the structural change. The cells treated with IG1 showed a thin cell wall with a rough edge compared to the control cell (Figure 5B), suggesting a disruption of the cell wall, whereas the cell membrane seemed intact.

## 4. Discussion

As a naturally occurring sesquiterpene *o*-quinone compound, several studies have highlighted the biological efficacy of mansonone F against Gram-positive bacteria, particularly MRSA. However, the limited availability in nature and the difficulties involved in its separation make it difficult to obtain in high quantities. Moreover, its activity is not significantly better than that of vancomycin and its antibacterial mechanism is still not well understood, which has promoted the exploration of the synthesis and bioactivity study of its analogs. In the present study, we obtained a mansonone F analog IG1 bearing a structure with 9-substituted, 3-substituted and opened-C rings [19], and this compound had good anti-*S. aureus* activity, so it was selected for in-depth study. The susceptibility test results showed that the antibacterial activity of IG1 against *S. aureus* including MRSA was superior to that of vancomycin, especially under high-inoculation concentration conditions, with am MIC range of 0.5~4 μg/mL VS. 4~16 μg/mL, which suggested that there are differing antimicrobial properties or mechanisms of action between IG1 and vancomycin.

Previous studies have shown that infections caused by *S. aureus* usually respond as well to bacteriostatic agents as to bactericidal ones [20,21]. To evaluate the antibacterial properties of IG1, we further conducted time–kill studies. Given that vancomycin is a rather weak cell-killing antibiotic and moxifloxacin is a rapid one [22,23], both agents were used as controls. Time–kill curves showed that the pattern of the change in viability after IG1 treatment was very similar to that of linezolid treatment, as shown in the report by Frech DL [24], and the latter has a bacteriostatic nature. In contrast, bacterial killing by vancomycin or moxifloxacin were obviously time- and concentration-dependent. Furthermore, the susceptibility of MRSA to IG1 was affected little by the inoculum concentration over a wide range from 1 × 10^5^ to 1 × 10^9^ CFU/mL, which undoubtedly showed the potentially powerful antimicrobial activity of IG1 against MRSA.

Mansonone F derivatives have been proven to be strong inhibitors against topoisomerase II, which may be one of the major targets for their antitumor action [19]. Another study further confirmed that cells treated with the mansonone F derivative IG3 for 24 h resulted in a dose-dependent accumulation of DNA in the sub-G1 phase and induced apoptosis [18]. To confirm whether IG1 also has effects on DNA replication in MRSA, we examined the DNA content and fluorescence intensity distribution of the PI-stained cells, finding that DNA content decreased significantly after IG1 treatment (Figure 4A). Further quantitative statistical analysis revealed that IG1 led to a time-dependent decrease in intracellular DNA content (Figure 4B). As a control drug with a clear antibacterial mechanism against cell wall synthesis, vancomycin treatment caused little change in DNA content, which suggests that IG1 may have a different mechanism of action on MRSA. The results of TEM image analysis further revealed that affecting DNA synthesis was likely to be an important mechanism for the anti-MRSA activity of IG1, since it was very difficult to find dividing cells in the IG1-treated MRSA strain.

TEM analysis is widely used for bacterial morphological examinations. The results of TEM implied that the mechanism of action of IG1 against MRSA strains may be related to direct or indirect impairment of cell wall synthesis. Although IG1 could interfere with cell wall synthesis, the characteristics of its antibacterial activity were very different from those of vancomycin, which acts on the cell wall. The above results also suggest that it may be difficult for IG1 to be hydrolyzed or sequestered by *S. aurues*, and that it may function as a modification reagent and result in cell wall damage, either directly or indirectly. Overall, IG1, or even mansonone F and other analogs or derivatives, can exert antibacterial activity against MRSA by potentially disrupting the cell wall and inhibiting the replication of DNA. This result will be helpful in order to uncover whether the potential antimicrobial mechanism of the activity of IG1 against *S. aureus* or MRSA is comparable or slightly stronger than vancomycin in vitro.

## 5. Conclusions

In the present study, we investigated the antimicrobial activity, antimicrobial properties, and antimicrobial mechanism of the mansonone F analog IG1 against *S. aureus*, including MRSA. To the best of our knowledge, this was the first ever study to evaluate both these properties and mechanisms. Based on the obtained results, it can be concluded that IG1 is a potentially effective drug against *S. aureus*, including MRSA, and that it may use multiple antimicrobial mechanisms.

## Figures and Tables

**Table 1:** Antimicrobial susceptibility of *S. aureus* to IG1 and vancomycin.

Strain	Numbers	MIC (μg/mL)			
		1 × 10^5^ CFU/mL ^1^		1 × 10^9^ CFU/mL	
		IG1	Vancomycin	IG1	Vancomycin
MSSA	17	0.25~2	0.5~2	0.5~4	4~16
MRSA	8	0.5~2	0.5~2	0.5~4	4~16
ATCC 25923	1	0.5	2	0.5	8

^1^ Inoculum concentration as referenced by CLSI.

## Data Availability

Not applicable.
